# Copper sulfide and zinc oxide hybrid nanocomposite for wastewater decontamination of pharmaceuticals and pesticides

**DOI:** 10.1038/s41598-022-22795-9

**Published:** 2022-10-28

**Authors:** Reem Mohammed, Mohamed Eid M. Ali, Ehsan Gomaa, Mona Mohsen

**Affiliations:** 1 Physics Department, Faculty of Science, Aim Shams University, Abbassia, P.O. 11566, Cairo, Egypt; 2 Water Pollution Research Department, Institute of Environmental Research and Climate Changes, National Research Centre, El-Buhouth St., Dokki, P.O. 12622, Cairo, Egypt

**Keywords:** Environmental sciences, Materials science, Nanoscience and technology, Renewable energy

## Abstract

In this work, hybrid nanocomposites of CuS QDs @ ZnO photocatalysts are fabricated through a facile microwave-assisted (MW) hydrothermal method as a green preparation process. The prepared photocatalysts (PCs) are employed under simulated sunlight (SL) for the degradation of ciprofloxacin, ceftriaxone, ibuprofen pharmaceuticals, methylene blue dye, and 2,4,5-trichlorophenoxyacetic acid (2,4-D) pesticide. The prepared photocatalysts are characterized in detail using several compositional, optical, and morphological techniques. The influence of the CuS (QDs) wt. % on morphological, structural, as well as photocatalytic degradation efficiency have been investigated. The small displacement between the (107) plane of CuS and the (102) plane of ZnO can confirmed the existence of lattice interaction, implying the formation of p-n heterojunctions. TEM and XRD results demonstrated that the CuS QDs are established and uniformly decorated on the surface of ZnO NRs, confirming the forming of an efficient CuS QDs @ ZnO heterojunction nanostructures. The CuS QDs @ ZnO hybrid nanocomposites showed enhancement in crystallinity, light absorption, surface area, separation of e–h pair and inhibition in their recombination at an interfacial heterojunction. In addition it is found that, 3 wt% CuS QDs @ ZnO has the foremost influence. The results showed improvement of photocatalytic activity of the 3% CuS QDs @ ZnO hybrid nanocomposite as compared to the bare ZnO nanorods. The impressive photocatalytic performance of CuS @ ZnO heterostructure nanorods may be attributed to efficient charge transfer. The prepared CuS QDs @ ZnO hybrid nanocomposites exhibited 100% removal for MB dye, after 45 min, and after 60 min for ibuprofen, ciprofloxacin pharmaceuticals, and 2.4.5 trichloro phenoxy acetic acid pesticide with the catalyst amount of 0.2 g/L. Although 100% removal of ceftriaxone pharmaceutical acheived after 90 min. In addition CuS QDs @ ZnO hybrid nanocomposites exhibited complete removal of COD for ibuprofen, ceftriaxone pharmaceuticals and 2.4.5 trichloro phenoxy acetic acid pesticide after 2 h with no selectivity. Briefly, 3% CuS QDs@ZnO hybrid nanocomposites can be considered as promising photoactive materials under simulated sunlight for wastewater decontamination.

## Introduction

Industrial wastewaters contain various toxic and carcinogenic compounds (e. g., synthetic organic dyes, pharmaceuticals, pesticides, and textile wastes) that endangers the environment's delicate balance. These wastes cause a hazard in the environment if released without treatment^1,2^. Synthetic organic dyes are widely used in several industries i.e. textiles, paper, plastic, leather, food, and others due to their higher stability and non-bio-degradable nature. These industries result in the production of effluent, which contains carcinogenic and hazardous dyes, polluting the water and providing it unfit for human consumption. During the dyeing process, these effluents are discharged into lakes, rivers, or groundwater, which leads to very serious environmental problems, due to their good stability under ambient conditions^3,4^.

Methylene blue (MB), which is commonly used to colour silk, wool, cotton, and paper, is one of the most expensive materials in the dye industry. Excessive MB exposure can result in difficulty breathing, mental confusion, eye burns, methemoglobinemia, sweating, nausea, vomiting, and profuse sweating^5^. Consequently, it is critical for the environment to remove such synthetic dyes before they are released into bodies of water. Pharmaceuticals are widely used to resist disease and enhancing the health of humans, animals, and other ecosystems. They can be discharged into environment through different pathways such as agriculture manure, animal excreta, and effluents from pharmaceutical industries and hospitals. Moreover, even in low doses, the release and persistence of these pharmaceuticals into the environment from several sources provide a potential risk to the health of organisms, mainly humans. Among these pharmaceuticals antibiotics have gained significant importance which is used in employed to treat infections caused by different bacteria, veterinary care, medicine, and farming^6^. Ciprofloxacin and ceftriaxone are antibiotics that are commonly used to treat infections caused by various bacteria. The excess use of antibiotics has created adverse effects on human health due to polluted water resources as they found in hospital discharges and industrial wastewater from pharmaceutical sources^7–9^. Antibiotics in the environment cause bacteria to become resistant to them, rendering them ineffective for a variety of infections and posing a risk to human health^10^. It is therefore critical to effectively remove these antibiotics from wastewater.

In addition, several types of pesticides generate pollution of air, soil, groundwater, and surface water that is damaging to human well-being as they are discharged into the atmosphere because of runoff from farming and civic areas^11^. Pesticides are the second major potable water contaminant and pose the greatest threat^9,12^. As a pesticide, it should be harmful to the intended pests but not to non-intended species like human beings and many other creatures. However, it is toxic to both envisioned and non-envisioned species.

2,4,5-trichlorophenoxyacetic acid (2,4,5-T) is a toxic derivative of 2,4,5-trichlorophenol and one of the most widely used pesticides in agriculture. Consumption of 2,4,5-T can cause brain and central nervous system damage in humans. Moreover, it is considered to be less biodegradable than similar phenoxy acetic acid pesticides (e.g., 2,4-dichlorophenoxyacetic acid, (2,4-D) due to the extra chlorine atom on the aromatic ring^13,14^.

Traditional treatment methods cannot make significant changes in their chemistry or concentration since they are based on transfer from one phase to another, leaving pollution problem solutions for further generations. A variety of physico-chemical methodologies are proposed for the removal of different organic pollutants from industrial waste, such as membrane bioreactors^15^, electrochemical treatment^16^, nanofiltration^17^, biological methods^18^, coagulation/flocculation^19,20^, adsorption^21^, chemical oxidation^22^, reaction with ozone^23^, and membrane processes^24^. However, several technical problems appeared. In the coagulation or flocculation method, pollutants are transferred from the liquid phase to a solid phase, causing secondary pollution. Adsorbent regeneration is an expensive and time-consuming process. Methods like reactions with ozone or hydrogen peroxide are very expensive and difficult to implement. The resulting by-products of chemical degradation may be themselves colored and/or even toxic^25^. Biological degradation of dyes is cost-effective, environmentally friendly, and does not produce huge amounts of sludge, but is selective, time-consuming, and thus not suitable for most of the dyes. Membrane technology is also used for removing dyes from wastewater. Due to the smaller particle diameter, the required membrane pore size should be in the ultrafiltration/nanofiltration range, which necessarily involves high pressures. However, the major problem in membrane-based separation processes is the decline in the flux and subsequent fouling of the membrane^26^. Recently, one of the most cost-effective and eco-friendly processes used for the degradation of organic effluents is the advanced oxidation process, particularly heterogeneous photocatalysis. This process depends on the generation of hydroxyl radicals (^·^OH) and reactive oxygen species (ROS) that are very active oxidizing agents for degrading different organic compounds under irradiation. ZnO is a n type semiconductor-based photocatalyst that can yield electron hole pairs when exposed to UV light. Because of its photostability, low operating temperature, high chemical stability, water insolubility, and non-toxicity, ZnO has been found to be the most efficient photocatalytic activity. Thus, it is frequently used for such purposes as mentioned in our previous publications^27,28^. However, due to its wide band gap (3.2 eV), it does not work when exposed to visible light. To improve ZnO's photocatalytic activity, various approaches have been applied to narrow the band gap and inhibit recombination of photogenerated electron–hole pairs, including conjugating with other semiconductor materials or doping with nonmetals/transition metal ions^28^. In the last several decades, much research has been focused on the coupling of ZnO semiconductor with its wide band gap with several other narrow band gap semiconductors to form heterostructures, such as SnS, MoS_2_, and ZnS^29–32^. Chen et al. reported that the fabrication of ZnO/Bi_2_WO_6_-CC composite by the thermal decomposition technique can be used as photodegradation of MB, and found that it can degradation about 96% of MB after 100 min^33^. Polymeric CA-ZnO nanocomposite membranes are fabricated using the phase inversion method and sono-hydrothermal synthesis technique by Abu-Dalo et al. Abu-Dalo et al. showed about 75% degradation of MB dye^34^.

Wolski demonstrated the degradation of ciprofloxacin (CIP) over CeO_2_/ZnO nanocomposites which synthesis by a simple co-precipitation method and reached to 60% degradation after 60 min^35^. Altynbaeva et al. revealed the use of Cu_2_O/ZnO @ PET composite membrane for the degradation of carbendazim pesticide^36^. Ebrahimi, Roya, et al. has been presented the photocatalytic degradation of 66.2% of 2,4-D by using Mn-doped zinc oxide/graphene nanocomposite under LED radiation after 120 min^37^. Zhu, Wenli, et al. demonstrated the degradation of RhB dye over CuO–CuS–ZnO–ZnS nanocomposite, more than 90% of RhB dye are removed under the simulated sunlight^38^. In our previously reported work, we synthesized ZnO photocatalyst for photocatalytic degradation of MB, CIP on solar simulator light exposure and found that ZnO catalyst exhibited the advanced dye degradation efficiency 83%, 60% respectively after 45 min^27,28^.

Nevertheless, to the best of our knowledge, up to now there are few articles about ZnO NRs mixed with CuS NP to form CuS @ ZnO nanocomposites. And concerning such CuS QDs @ ZnO nanocomposites material to remove (MB dye, ibuprofen, ceftraxone, ciprofloxacin, and 2,4-D pesticide from simulated wastewater have not been reported yet.

This nanocomposite facilitates the catalyst to absorb along a wide range of the solar spectrum. Furthermore, the band gaps of both coupled semiconductors are located in various band edge positions, which enhances charge transfer at the interface and delays electron–hole pair recombination, resulting in improved photocatalytic performance.

This work aims to enhance the photocatalytic degradation efficiency of the previously prepared ZnO nanorods using CuS QDs, by forming CuS QDs @ ZnO hybrid nanocomposite through a simple microwave hydrothermal method. The photocatalytic properties of the CuS QDs @ ZnO hybrid nanocomposite are investigated against ciprofloxacin, ceftriaxone, ibuprofen pharmaceuticals, methylene blue dye, and 2,4,5-trichlorophenoxyacetic acid pesticide (2,4-D) degradation under solar simulating irradiation. Consequently, the process of improving the solar light-induced efficiency of CuS QDs @ ZnO hybrid nanocomposite has been discussed in detail in this work.

## Experimental work

### Materials and reagent

Zinc nitrate hexahydrate (Zn (NO_3_)_2_·6H_2_O), hexamethylenetetramine (HMT), are supplied by Merck Chemicals Company, Germany.

Copper acetate monohydrate CA (Cu (CH_3_COO)_2_) powders (≥ 99.0% in purity), are supplied by Sigma Aldrich, Germany. Thiourea TU (CH_4_N_2_S) is supplied by Fluka Chemicals Company, Germany.

All the chemicals employed are of analytical grade and are applied without further purification. The solvent utilized in all the experimental procedures is deionized water.

As well as, reagent grade methylene blue (MB) is supplied by LOBA Chemie, India. 2,4,5-trichlorophenylacetic acid (C_8_H_5_F_3_O_2_), Ibuprofen (C_13_H_18_O_2_), and ceftriaxone (C_18_H_18_N_8_O_7_S_3_), pharmaceutics are supplied by Sigma-Aldrich, Germany.

### Preparation of CuS quantum dotes (QDs) and CuS QDs @ ZnO hybrid nanocomposites photocatalysts

#### Preparation of CuS quantum dots (QDs)

In a typical microwave hydrothermal synthesis, 100 mmol of copper acetate monohydrate [Cu (CH_3_COO)_2_] is dissolved in 100 mL of deionized water (DW) to form a clear blue solution, then after complete dissolution, under vigorous stirring for 1 h., 100 mmol of thiourea (TU) powder is added. The prepared solution immediately turned to greenish-brown. To enable proper mixing, the solution is repeatedly stirred for 1 h. The solution is then transferred to a microwave oven at full power (Stainless Steel MW oven, 120 Volts, 1000 W, 200 °C) for 6 min (2 min on and 30 s off, repeat for 3 cycles). The black solid products of CuS QDs are then collected by filtration on filter paper (14–18 μm), washed with distilled water several times each, and dried in an oven at 60 °C for 6 h before further characterization.

#### CuS QDs @ ZnO hybrid nanocomposite preparation

**Figure 1:**
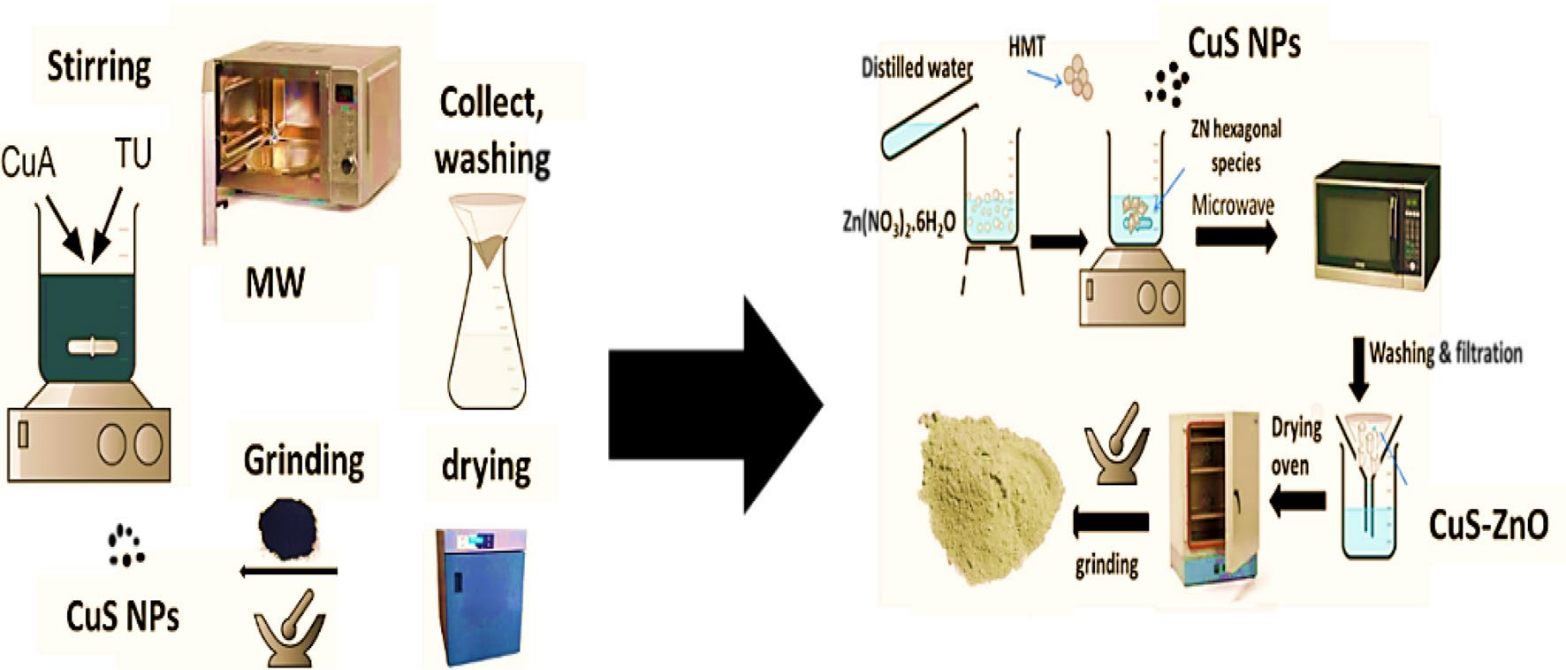
Schematic proposed diagram for the preparation of CuS QDs and CuS QDs @ ZnO hybrid nanocomposites.

The CuS QDs @ ZnO hybrid nanocomposite photocatalysts are fabricated using a simple microwave-assisted hydrothermal reaction. Typically, as illustrated in Fig. 1, 0.1 M of Zn ((NO_3_)_2_.6 H_2_O) is stirred in 50 ml of DW for half an hour, and to this solution, 0.1 M of HMT is added. After continual stirring for 2 h, in another beaker, different ratios of the last prepared CuS QDs (0.5, 1, 3, 5, 7, and 9%) are dispersed in 50 ml of deionized water for 1 h. The two solutions are mixed with vigorous stirring for another 1 h. The reaction mixture is poured into a 100-ml beaker and placed in the microwave for 2 min (5 cycles). The grey precipitate thus obtained is cleansed and separated by filtration with distilled water. CuS QDs @ ZnO hybrid nanocomposites are prepared by drying the obtained sample in an oven at 70 °C for 10 h. For comparison, ZnO NRs are prepared with the same procedure without the addition of CuS nanoparticles. The white precipitate obtained is cleansed and separated by filtration with distilled water. ZnO is prepared by drying the obtained sample in an air oven at 70 °C for 10 h. The resultant CuS QDs @ ZnO hybrid nanocomposites are labeled x%—CuS QDs @ ZnO, where x is the weight ratio of CuS to ZnO. Table 1 illustrates the preparation conditions of different CuS QDs @ ZnO hybrid nanocomposites.

**Table 1:** The preparation condition for preparation of different CuS QDs @ ZnO hybrid nanocomposites.

Sample no	Microwave irradiation time (min)	Wt% of CuS QDs @ZnO Wt.%
0.5 wt% CuS QDs @ ZnO	6	0.5
1 wt% CuS QDs @ ZnO	6	1
3 wt% CuS QDs @ ZnO	6	3
5 wt% CuS QDs @ ZnO	6	5
7 wt% CuS QDs @ZnO	6	7
9 wt% CuS QDs @ZnO	6	9

### Characterization

A PANalytical X'PertPRO diffractometer with CuK (= 1.54059) incident radiation, powered at 40 kV and 30 mA, is used for X-ray diffraction (XRD) analysis.The patterns are collected in the range of 10 ^o^ –70° with a step of 0.03. The crystallite size of the prepared samples is calculated from the XRD line-broadening measurement from the Debye–Scherrer equation^39,40^, as follows:

1 $$Crytallite\, Size=\frac{0.89\lambda }{\beta \mathrm{cos}\theta }$$

where $\lambda$ is the wavelength, (Cu K_α_) is the full width at the half maximum of the ZnO (101) line and is the diffraction angle. The Fourier transform infrared (FTIR) absorption spectra of the samples are collected at room temperature in the range of 400–4000 cm^−1^ by an infrared spectrophotometer, the BRUKER VERTEX 70, with a resolution of 4 cm^−1^ using the KBr disc technique. The elemental analysis and morphology of the samples are examined by energy-dispersive X-ray spectroscopy (EDX) and scanning electron microscopy (SEM) respectively, using a field emission scanning electron microscope ((FE-SEM) (QUANTA FEG250 operated at 20 kV). The nanostructure, degree of crystallinity, diffraction patterns, composition, and average particle size, are studied under a high-resolution transmission electron microscope (TEM) (JEOL JEM-2100) with a high resolution of 0.19 nm at 200 kV (LaB6). UV–Visible diffuse reflectance spectroscopy (Shimadzu UV-2401 PC spectrophotometer equipped with diffuse reflectance accessories) is used to investigate the optical properties of the materials (UV–visible spectra and the band gap energy), at a resolution of 2 nm over the wavelength range between 190 and 1100 nm. The photoluminescence (PL) spectrum of the prepared photocatalysts is recorded in the spectral range of 220–625 nm at room temperature on a Perkin Elmer LS 3B spectrophotometer at the 320 nm excitation wavelength. The specific surface area, pore size, and pore volume of the prepared photocatalysts are estimated using a Sorptomatic (Model-1990) instrument at 77 K.

### Photocatalytic experiments

**Figure 2:**
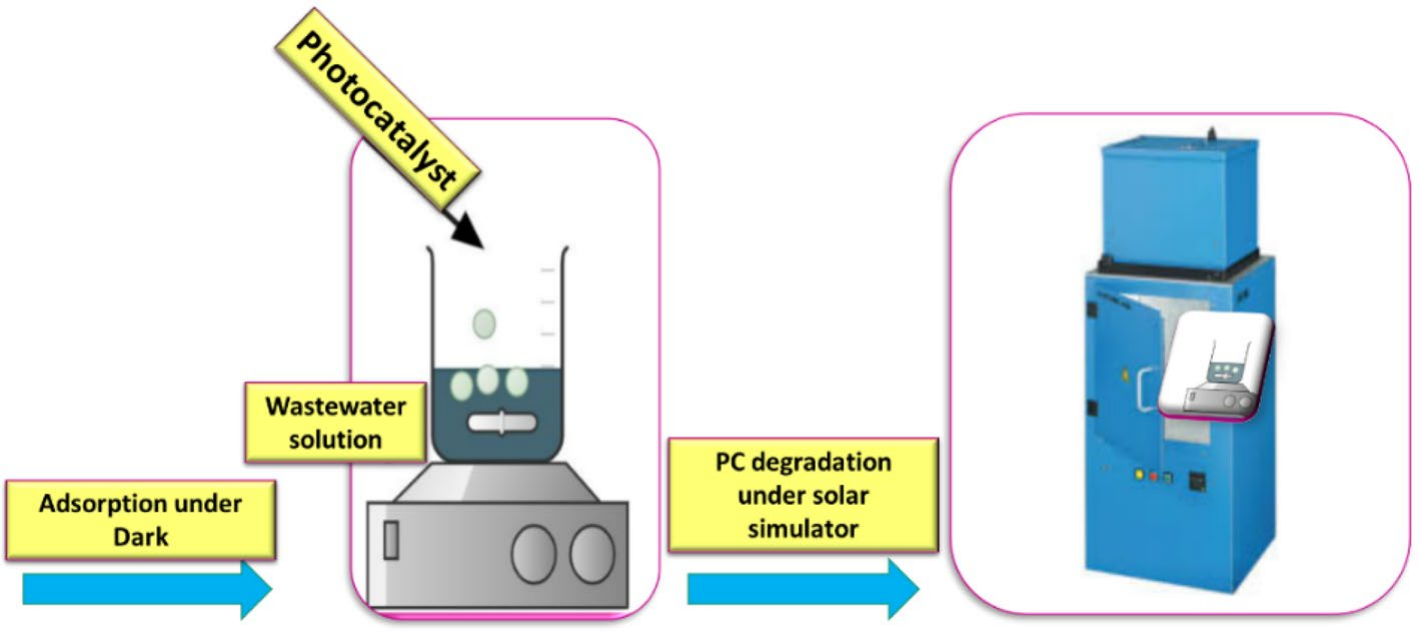
Schematic proposed diagram of the photocatalytic degradation process.

A solar simulator is used as a laboratory photocatalytic unit for the batch experiments. The schematic diagram of the experimental system used is revealed in Fig. 2. It consists of an external stirrer with a variable speed that is put into the closed solar simulator photoreactor. The solar simulator is an simulating the solar energy an irradiation chamber for solar simulation, UVA CUBE 400, 400 W, ranged from 300 nm to 1200 nm. Certain weight of the synthesized photocatalysts is dispersed in the simulated wastewater under continuous stirring at ambient temperature. Adjusting pH value of solutions is carried out by using HCl (0.2 M) and NaOH (0.2 M).

In the photo degradation process, 0.2 g .L^−1^ of photocatalyst (PC) is suspended in an aqueous solution of 20 ppm of pollutants. Before irradiation, the solution is stirred for 30 min in the dark to reach the adsorption–desorption equilibrium. Then, the solar simulator is turned on; and a sample solution is taken every 15 min. Then, the withdrawn suspensions are filtered with a syringe filter (Whatman, 0.45 µm). The concentrations at different times are measured utilizing the spectrophotometer (UV–Vis spectrophotometer, a JASCO-V-730).

The degradation efficiency is measured according to Eq. (2):

2 $$Degradation\, \%=\frac{{C}_{0}-{C}_{t}}{{C}_{0}}\times 100$$

where C_0_ and C_t_ are the concentrations of the waste before and after different irradiation times, respectively.

The COD removal (mineralization rate) is determined using Eq. (3).

3 $$\mathrm{Removal\, COD\, \% }=\frac{{C}_{0}-{C}_{t}}{{C}_{0}}\times 100$$

where C_0_ is initial COD in ppm and C_t_ is COD in ppm at time interval t.

## Results and discussion

### Structure analysis

**Figure 3:**
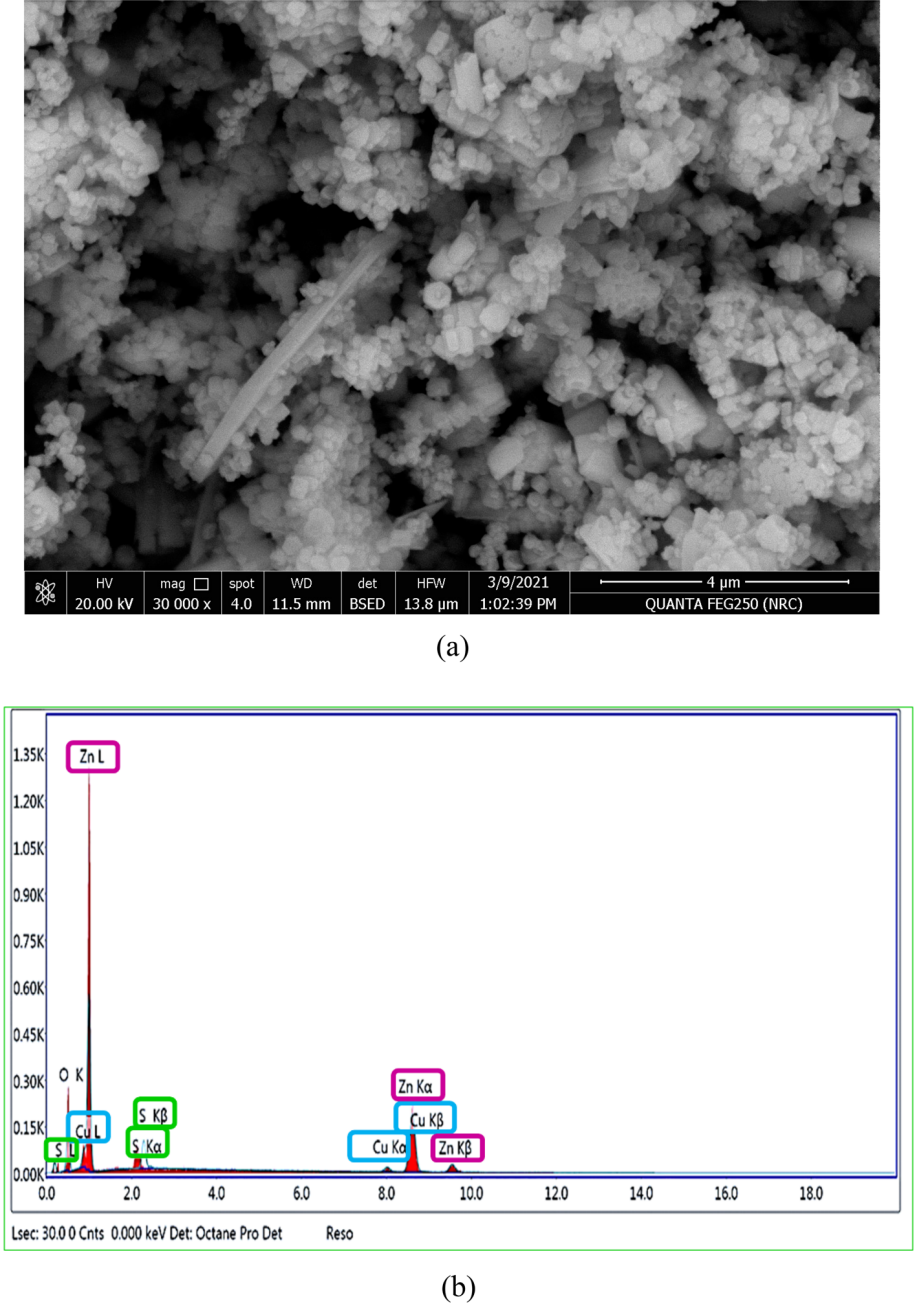
(**a**) SEM image of a hybrid nanocomposite containing 3% CuS QDs @ ZnO. (**b**) EDX spectrum of a 3% CuS QDs @ ZnO hybrid nanocomposite.

The microstructure and morphological analysis of 3% CuS QDs @ ZnO hybrid nanocomposite are carried on Field emission scanning electron microscopy (FESEM) which are presented in Fig. 3a. As illustrated in the figure that 3% CuS QDs @ ZnO is mainly composed of ZnO nanorod with rough surface covered with CuS nanoparticles which are different from the pristine ZnO in our previous publication^27,28^. It suggests that the CuS has a slight effect on the morphology and structure of ZnO NRs.

The electron-dispersive X-ray spectroscopy (EDAX) analysis of CuS QDs @ ZnO nanocomposite confirms the presence of the elements Cu, S, Zn, and O in the sample and formation of hybrid nanocomposite as illustrated in Fig. 3b, Table 2. The absence of any other elemental peaks revealed the prepared sample's high purity.

**Table 2:** EDX data of 3% CuS QDs @ ZnO hybrid nanocomposite.

Element	Weight %	Atomic %	Net Int	Error %
O K	18.15	46	86.32	10.95
S K	4.9	6.2	63.36	12.29
Cu K	4.13	2.63	16.2	20.86
Zn K	72.82	45.17	216.59	3.81

**Figure 4:**
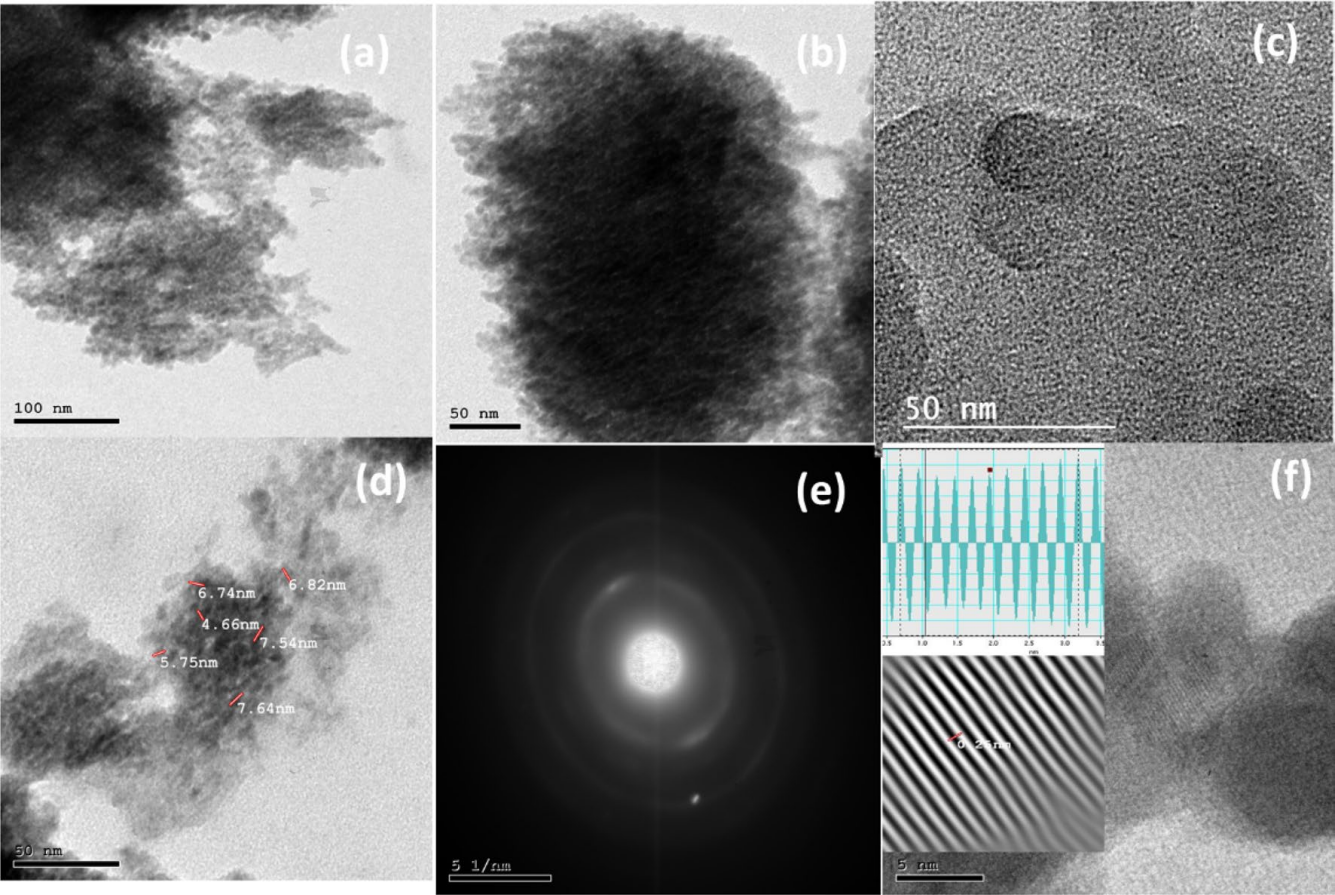
(**a**–**d**) Transmission electron image (TEM) of CuS QDs. (**e**) The selected area electron diffraction pattern in a specific region of a CuS QDs. (**f**) The lattice plane spacing of a CuS QDs.

Transmission Electron Microscopy (TEM) characterization is used to investigate the chemical composition, structure, and interactions of CuS (QDs) and CuS QDs @ ZnO hybrid nanocomposite. The TEM images of as synthesized CuS QDs and CuS QDs @ ZnO hybrid nanocomposite are revealed in Fig. 4.

**Figure 5:**
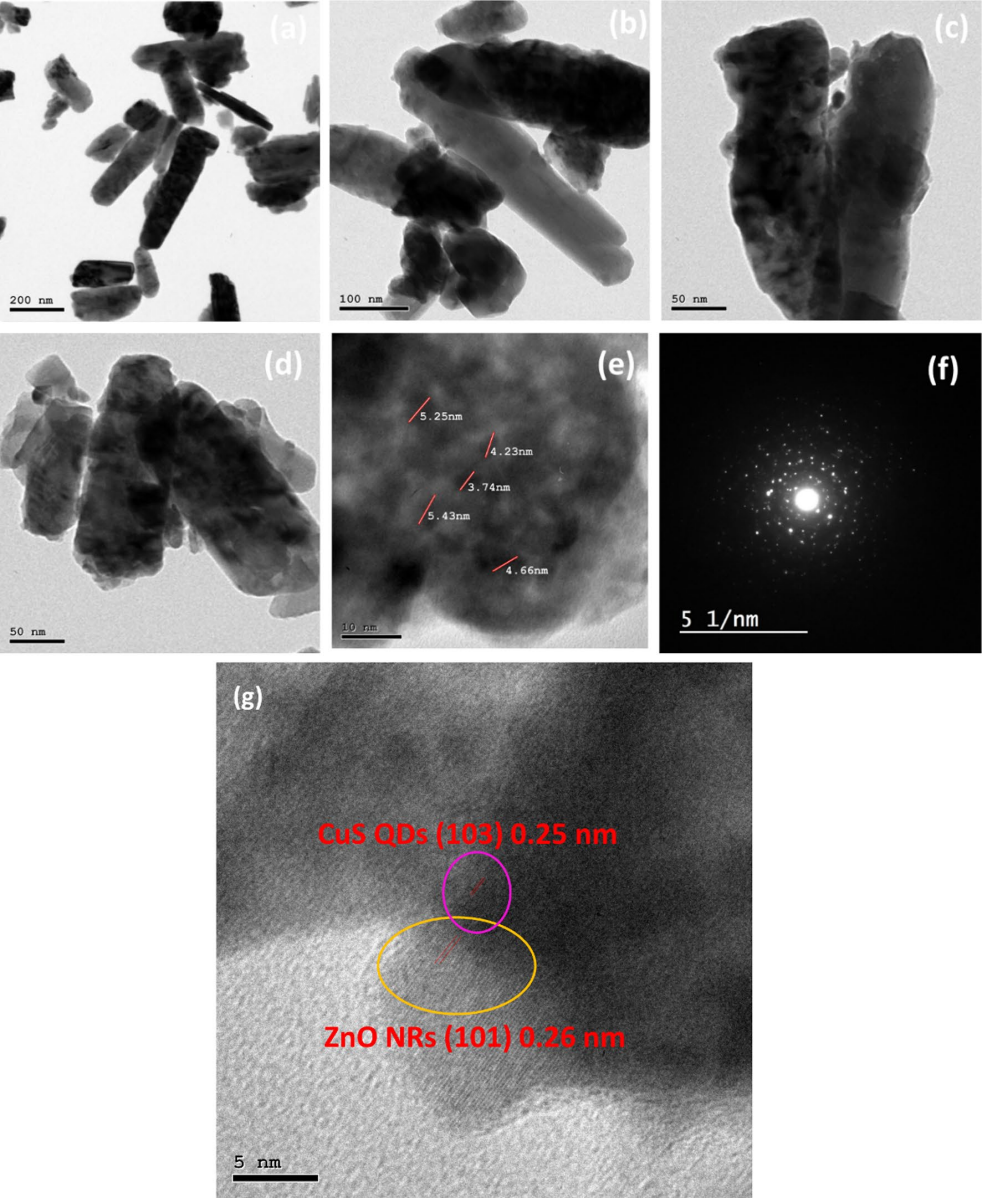
(**a**–**e**) Transmission electron image (TEM) of 3% CuS QDs @ ZnO hybrid nanocomposite. (**f**) The selected area electron diffraction pattern in a specific region of a 3% CuS QDs @ ZnO hybrid nanocomposite. (**g**) The lattice plane spacing of CuS QDs @ ZnO hybrid nanocomposite ( CuS QDs and ZnO NRs).

The TEM and HRTEM images of the synthetic CuS particles (Fig. 4a–d) reveals that the CuS particles is a QDs with a sphere-like morphology of an average diameter of 4 nm. The selected Area Electron Diffraction (SAED) pattern of CuS QDs is illustrated in Fig. 4e. Furthermore, as determined from the high-resolution TEM (HRTEM) image (Fig. 4f), the interplanar distance of CuS QDs is 0.25 nm. (Fig. 5a–e) of the CuS QDs @ ZnO hybrid nanocomposite provides more information about the uniform bonding interaction of hexagonal CuS on the surface of ZnO NRs. Furthermore, it demonstrates that the CuS QDs are established and uniformly decorated on the surface of ZnO NRs, confirming the forming of an efficient CuS QDs @ ZnO heterojunction nanostructures. This active heterojunction facilitates in photogenerated charge transfer during the photocatalytic phase, which causes pollutant mineralization. The nearer image (Fig. 5b–d) provides additional insight into the shape and size of ZnO NRs, demonstrating that ZNRs are unbroken, homogenously distributed, and have an average diameter of 100 ± 10 nm. In addition, The TEM image clearly indicates that the as obtained spherical shaped CuS nanoparticles with average particle sizes ranging from 1.5 to 7 nm are uniformly distributed on the ZnO NRs. The selected Area Electron Diffraction (SAED) pattern in Fig. 5f stated clearly the crystalline nature of CuS QDs @ ZnO by the bonding interaction between CuS and ZnO as it is different from that of bare CuS QDs (See Fig. 4e) which confirm the formation of nanocomposite. The high-resolution TEM (HRTEM) image (Fig. 5g) shows that the as prepared CuS QDs on the ZnO NRs have interplanar distances of 0.25 and 0.22 nm, which coincide to the d spacing of (103), (101) planes, respectively.

**Figure 6:**
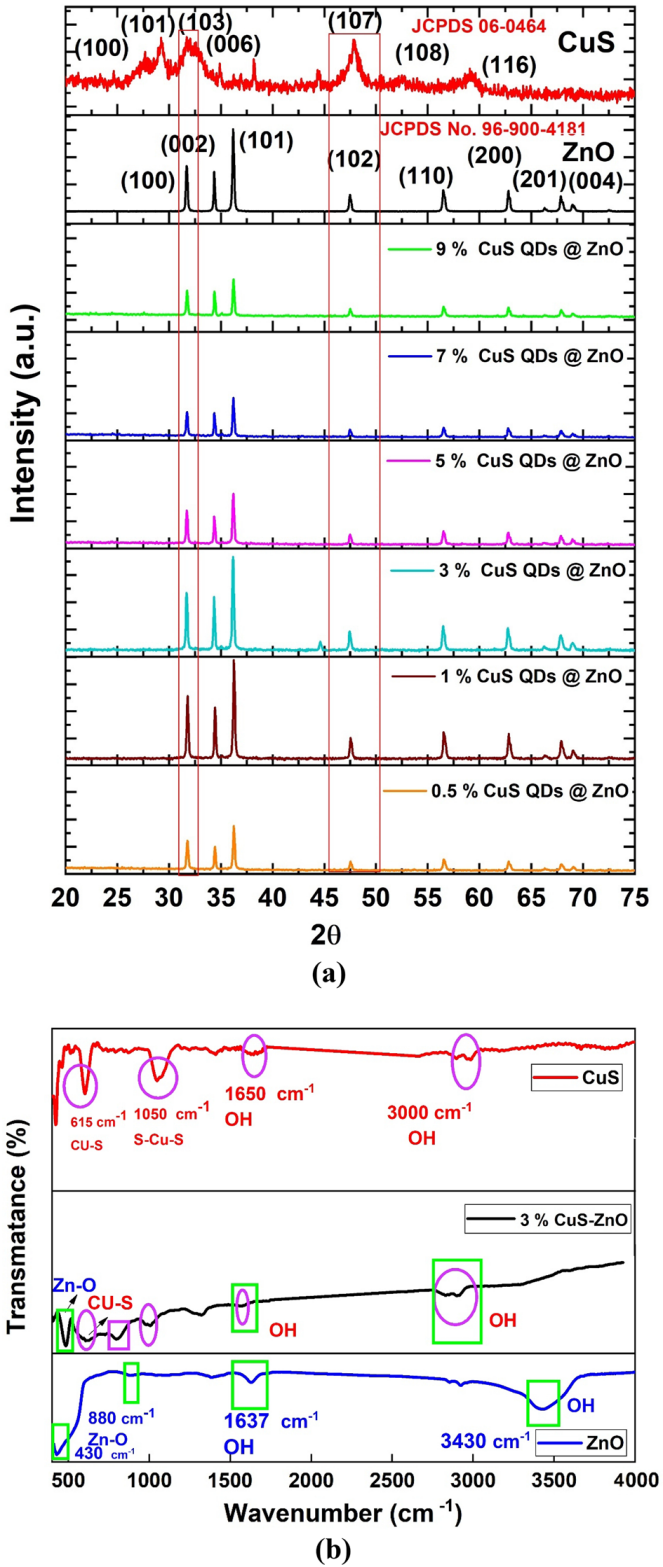
(**a**) XRD patterns of bare ZnO, CuS QDs, CuS QDs @ ZnO hybrid nanocomposites with different CuS QDs content wt%. (**b**) FTIR spectra bare ZnO, CuS QDs, 3% CuS QDs @ ZnO hybrid nanocomposites.

The phase purity, structure, composition, and crystallinity of the synthesized materials are determined using powder XRD measurements. Figure 6a shows the X-ray diffraction (XRD) patterns of pure CuS QDs, pure ZnO NRs, and x % CuS QDs @ ZnO hybrid nanocomposite where x is the weight ratio of CuS QDs to ZnO NRs.

The XRD pattern of CuS QDs demonstrated that the products grown are CuS NPs with cubic structure. All the diffraction peaks can be assigned to the pure covellite hexagonal phase (JCPDS 06-0464). It can be observed that CuS QDs have well-defined characteristic diffraction peaks with 2θ values of (27.7°, 29.3°, 31.76°, 32.7°, 47.8°, 52.3°, 59.2°) which are corresponding respectively to (100), (101), (103), (006), (107), (108), and (116) crystal planes of the preferential orientation of CuS NPs^41,42^. CuS QDs have relatively high crystallinity and the most intense peak at 2θ = 31.76 relates to (103) diffraction plane. The nanosized CuS particles cause the peak's relatively low intensity and relatively broadened width, revealing the presence of small crystallites^43,44^.

For the XRD patterns of ZnO, it is observed that all diffraction peaks can be indexed to the ZnO hexagonal wurtzite structure according to JCPDS No. 96-900-4181 of ZnO (Wurtzite hexagonal phase) with characteristic diffraction peaks at 31.7°, 34.42°, 36.24, 47.5°, 56.554°, 62.83°, 67.9°, and 69.01°, that are, corresponding to (100), (002), (101), (102), (110), (200), (201), and (004) planes, respectively. The characteristic peak of the (101) plane indicates the anisotropic growth of the crystallite and the formation of a hexagonal phase^27,28,45,46^.

On the other hand, it is worthwhile to notice that structural transformation of either ZnO NRs or CuS QDs is not observed and there is no diffraction pattern for any impurities, indicating that the preparation process is suitable^47^. It is also observed that, the d spacing of ZnO NRs and CuS QDs are 2.4 Å and 2.5 Å respectively which is inconsistence with TEM results.

Figure 6a shows the XRD pattern of the x % CuS QDs @ ZnO hybrid nanocomposite where the x is the weight ratio of (x = 0.5, 1, 3, 5, 7, and 9%) which is similar to that of ZnO NRs. The XRD pattern of ZnO in the CuS QDs @ ZnO hybrid nanocomposite remained consistent with that of ZnO (JCPDS No. 96-900-4181), thus suggesting that the ZnO is well maintained during the loading of CuS^48^. It is observed that all peaks are very sharp and intense which indicated the crystalline nature of the prepared nanocomposites and the addition of copper does not alter the crystalline phase of nanocomposite^49^.

From the CuS QDs @ ZnO XRD pattern, as the (107) and (103) planes of CuS appeared at 47.9° and 31.8°, respectively, and the (102) and (002) planes of ZnO appeared at 47.8° and 31.7°, it is observed that the CuS peaks at 31.78° and 47.64° are merged with ZNR peaks with a slight displacement. The small displacement between the (107) plane of CuS and the (102) plane of ZnO can confirmed the existence of lattice interaction, implying the formation of p-n heterojunctions^50,51^.

Additionally, for the nanocomposite patterns, the ZnO peak position slightly shifted towards higher 2θ value compared to pure ZnO. Moreover, no other additional characteristic diffraction peaks related to CuS QDs are observed. This may be owing to its low size, low concentrations, and its uniformly distribution along ZnO NRs. In the literature, similar observations about the absence of other characteristic CuS XRD signals in nanocomposites have been reported^39,52–54.^ Formation of nanocomposites is confirmed from TEM results, moreover other techniques will be checked to confirm the formation of nanocomposites^54,55^.

It is interesting to note that, the peak intensities of the composite samples increase gradually with an increase in the CuS QDs ratios from 0.5% to 3%. Further, when the amount of CuS is increased by more than 3%, a significant reduction of the peak intensities is observed indicating the limit of the uniform dispersion of CuS QDs showing aggregation in accordance with^56–58^. It can be deduced that with 3% CuS uniformly dispersed in the nanocomposite which is beneficial for the photocatalytic activity of the composite photocatalyst, increasing the CuS ratio leads to aggregation.

The average crystallite sizes of CuS QDs, ZnO nanorods, and CuS QDs @ ZnO hybrid nanocomposites are estimated using Scherer's equation^52^ and illustrated in Table 3. The approximate average crystallite sizes of CuS QDs and ZnO NRs are calculated to be 1.9 nm and 38.84 nm, respectively. It is found that the average crystallite size of 0.5% CuS QDs @ ZnO, 1% CuS QDs @ ZnO, 3% CuS QDs @ ZnO, 5% CuS QDs @ ZnO, 7% CuS QDs @ ZnO, and 9% CuS QDs @ ZnO are 39.7, 37.8, 36.5, 40.9, 42.5, and 46.3 nm, respectively. The results indicate that the crystallite size decreases with the addition and incremental the addition of the wt% CuS from 0.5 to 3%, then it increases with a further increase which affects the photocatalytic activity. The decrease in crystallite size is related to developing the larger grain boundaries which leads to a larger scattering effect or ZnO's drag force on grain growth and boundary motion. It can be concluded that the small amount of CuS QDs is uniformly dispersed all around the ZnO NRs system^57^.

**Table 3:** Average crystallite size for the prepared bare ZnO, CuS QDs, and their nanocomposites.

Sample	Crystallite size (nm)
CuS QDs	1.9
ZnO NRS	38.8
0.5% CuS QDs @ ZnO	39.7
1% CuS QDs @ ZnO	37.85
3% CuS QDs @ ZnO	36.5
5% CuS QDs @ ZnO	40.92
7% CuS QDs @ ZnO	42.52
9% CuS QDs @ ZnO	46.27

To examine the nature of bonds in the prepared materials, confirm the modifications carried out on ZnO NRs, and formation of the hybrid nanocomposites, the FTIR spectra of CuS QDs, ZnO NRs, and CuS QDs @ ZnO hybrid nanocomposites are collected in the range of 400–4000 cm^−1^ (Fig. 6b). The presence of Cu–S groups is indicated by stretching vibration peak detected at 615 cm^−1^ which shows the formation of CuS crystals^59–61^. Additional peak existed at 1050 cm^−1^ corresponding to the stretching vibration of S=O bonding^61,62^. The stretching and bending vibration of surface hydroxyl groups (OH group) is observed at 1650 cm^−1^ and 3000 cm^−1^ respectively confirms that water molecules are adsorbed on the CuS surface (H–O–H) in accordance with^57^.

From the FTIR spectrum of ZnO, the major peaks of ZnO have been observed at 430, 880, 1637, and 3430 cm^−1^. The peak at 442 cm^−1^ corresponds to Zn–O symmetric stretching vibration^63^ and the peak at 809 cm^−1^ is due to bending vibration of Zn–O in agreement with^42,63–65^. The peaks at 1635 cm^−1^ and 3422 cm^−1^ are due to O–H bending and stretching vibration mode respectively of the water molecule and surface hydroxyl groups. The existence of these bands indicates the presence of water molecules on the surface of the ZnO^27,28^. Therefore, the CuS and ZnO FTIR data indicate the successful preparation of CuS and ZnO without any impurity.

For the FTIR spectrum of 3% CuS QDs @ ZnO hybrid nanocomposite, the CuS and ZnO characteristic peaks appeared and shifted to 622 cm^−1^ and 484 cm^−1^ respectively. The presence of these two peaks confirmed the formation of CuS QDs @ ZnO hybrid nanocomposite^55^.

The peak at 3290 cm^−1^, 1596 cm^−1^ which is observed in the spectra of ZnO, CuS as well as in the nanocomposite and can be attributed to the stretching and bending of the H–O-H bond of adsorbed water^66–69^.

### Optical properties

**Figure 7:**
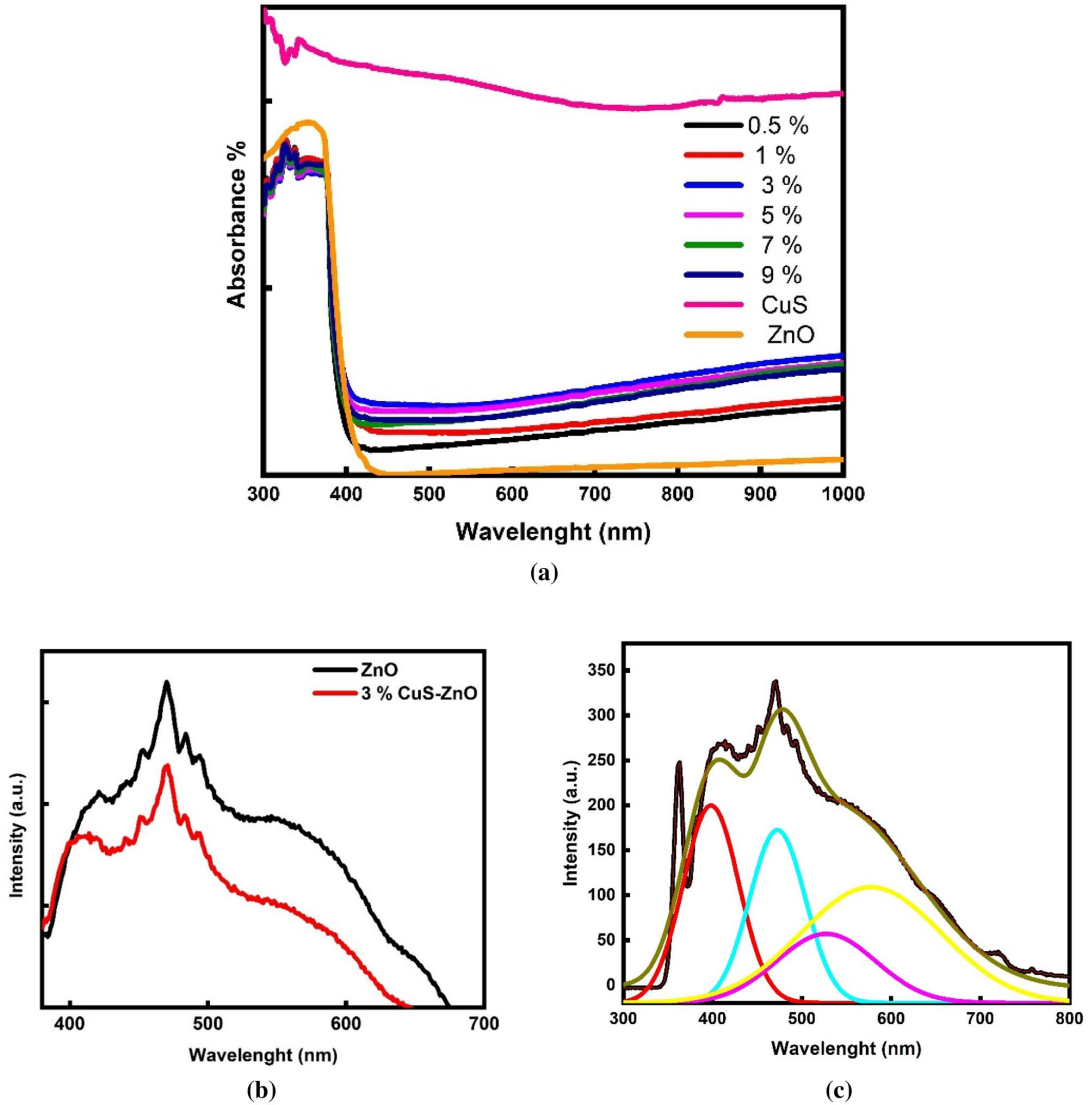
(**a**) UV–Vis (DRS) of bare CuS QDs and x wt% CuS QDS @ZnO hybrid nanocomposite. (**b**) Room-temperature PL spectrum of 3% CuS QDS @ZnO nanocomposite (**c**) Deconvolution of its emission spectra.

The optical absorption characteristics and electron–hole recombination rate of the as-prepared CuS QDs and CuS QDs @ ZnO hybrid nanocomposites are measured by UV–Vis diffuse reflectance spectroscopy (DRS) and PL spectroscopy, respectively. It can be seen that the CuS QDs prominently exhibits a broad absorption in the visible region (400–800 nm)^51,69^. The absorption edge of pure CuS QDs is approximately 1550 nm, as shown in Fig. 7a, which is corresponding to bandgap energy of 1.25 eV due to its black color^48,70^. In addition, ZnO NRs showed a broad peak of absorbance which is noted at about 397 nm, its corresponding bandgap is 3.12 eV^27,28,54^. The DRS profile of all the prepared nanocomposites samples exhibit a further increase in absorption range indicating that addition of CuS QDs to ZnO NRs has improved the visible light absorption as well thus facilitating the photocatalytic activity^62^.

It can be noted that, all nanocomposite spectra show the characteristic spectrum of ZnO but with absorption enhancement, showing that the bandgap values do not change significantly when CuS QDs are added^71^. Furthermore, increasing the CuS QDs content from 0.5% to 3% improves absorption then decreases with a further CuS % increase which is coincidence with XRD data which illustrate that 3% CuS DQs @ ZnO showed highly dispersibility. Improving solar spectrum absorption is the key to increasing photocatalytic activity. The absorption edges of 0.5% CuS QDs @ ZnO, 1% CuS QDs @ ZnO, 3% CuS @ ZnO, 5% CuS QDs @ ZnO, 7% CuS QDs @ ZnO, and 9% CuS QDs @ ZnO are shift to 400, 407, 420.5, 414.5, 414, and 411 nm, respectively, and the corresponding bandgap energies are 3.1, 3.05, 2.95, 2.995, and 3.1 eV, respectively^52,53^.

It is known that, smaller band gaps indicate lower energy required to induce efficient electron transfer. Therefore, CuS QDs modification leads to an increase in the surface electric charge of ZnO in the nanocomposite and enhance the fundamental process of electronic transformation during irradiationwhich helps in enhace the photocatalytic activity^51^.

Moreover, the optical bandgap of the prepared CuS QDs and CuS QDs @ ZnO hybrid nanocomposite can be deduced from the UV–Vis diffuse reflectance result according to the Kubelka–Munk function^72^. From the data, we conclude that all the systems follow the direct allowed transition, and the values of the corresponding bandgap are represented in Table 4 which is in close agreement with its visible light absorption ability^38,49^.

**Table 4:** Band gap values for different prepared x % CuS QDS @ ZnO hybrid nanocomposites.

Sample	Bandgap (eV)	Initial degradation rate (mg L^−1^ min^−1^)
ZnO	3.1	0.24
0.5% CuS QDS @ ZnO	3.1	0.36
1% CuS QDS @ ZnO	3.05	0.57
3% CuS QDS @ ZnO	2.95	0.86
5% CuS QDS @ ZnO	2.99	0.6
7% CuS QDS @ ZnO	2.995	0.4
9% CuS QDS @ ZnO	3.1	0.4

### Photoluminescence (PL) studies

The room temperature photoluminescence (PL) quenching spectra of the prepared samples are investigated to examine whether incorporating CuS nanoparticles onto ZnO NRs for the creation of heterojunction at interfaces affects the PL property and enhances the charge carrier's separation efficiency. The PL spectra measurements are done at the excitation wavelength 315 nm. As shown in Fig. 7b the PL peaks of the 3% CuS QDs @ ZnO showed lower intensity relative to that of bare ZnO NRs. These suggests that the heterostructures favored the efficient separation of photogeneration electrons and holes which help in transferring the photo-excited electrons generated by ZnO to CuS surface, prevent the charge recombination and accelerate transport and enhance lifetime of the photogenerated charge carriers. As the PL quenching reveals a lower electron–hole recombination rate and a higher electron-transfer rate in agreement with^57^. This result further confirmed that the CuS QDs are exfoliated and attached to the ZnO NRs surface during the microwave hydrothermal process, in agreement with^66^. Subsequently, decreasing recombination rate and enhancing visible light absorption of 3% CuS QDs @ ZnO might further respond to the effective utilization of photoexcited carriers that promoted the photocatalytic degradation reaction of organic dyes^73^.

The deconvolution of the 3% CuS @ ZnO defect emission band (Fig. 7c), which is centered at 478 nm, yields four defect bands. The energy difference between the bottom of the conduction band and the energy level of zinc vacancy is responsible for the strong violet peak at 406 nm. There is a defect peak at 472 nm (eV) that can be attributed to lattice defects related to oxygen and zinc vacancies, interstitial oxygen, and the transition between defects at grain boundaries and the valence band (VB). A broad emission peak in the 500–570 nm range, which can be attributed to electron transition from zinc interstitial or oxygen vacancy to the top of the valence band. Besides, a broad emission peak in the 500–580 nm range, which can be attributed to CuS band-edge emission, the blue-shift of the band-edge mission of small CuS nanoparticles, and complex defects of CuS nanostructures in accordance with^74^.

### BET analysis

In heterogeneous reactions, the specific surface area is proportional to the number of catalytic active sites. So, it is a key parameter in the photocatalytic activity of synthesised samples. The Brunauer–Emmett–Teller (BET) is used to calculate the specific surface area and the results have been presented in Table 5. The BET surface areas of the ZnO NRs and 3% CuS QDS @ ZnO hybrid nanocomposite are determined to be 124.2 and 150.5 m^2^ g^−1^, respectively. It can be concluded that the formation of the nanocomposite has increased the surface area of the ZnO, which is very suitable for the improvement of the degradation efficiency.

**Table 5:** The BET surface areas of the ZnO NRs, and 3% CuS QD @ ZnO hybrid nanocomposite.

Sample	Specific surface area (m^2^ g^−1^)
ZnO NRs	124.2
3% CuS QD @ ZnO hybrid nanocomposite	150.5

### Photocatalytic activity

The formation of hybrid nanocomposites containing CuS QDs can significantly change the absorption edge of ZnO NRs near the visible range, allowing absorption of a greater fraction of the solar spectrum and reduce the band gap energy. Additionally, the formation of these new states can prevent e^–^- h^+^ pair recombination as well as increase surface area thus improve the photocatalytic performance. However, increase the CuS QDs concentration higher than 3% results in the creation of mid gap states that act as charge recombination centers and degrade the photocatalytic activity.

#### Effect of CuS QDs @ ZnO hybrid nanocomposite on the photocatalytic degradation efficiency

**Figure 8:**
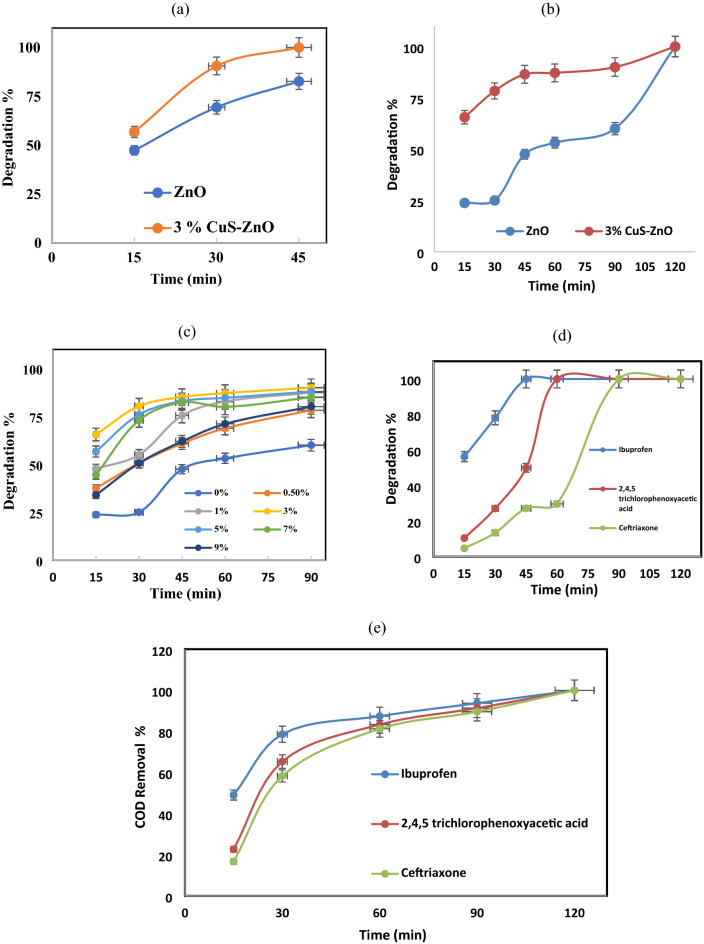
(**a**) Effect of 3% CuS QDs @ ZnO hybrid nanocomposite on degradation % of MB. (**b**) Effect of 3% CuS QDs @ ZnO hybrid nanocomposite on degradation % of ciprofloxacin. (**c**) Effect of CuS QDs ratio on degradation % of ciprofloxacin (**d**) Effect of 3% CuS QDs @ ZnO hybrid nanocomposite on degradation of different pharmaceuticals (**e**) Effect of 3% CuS QDs @ ZnO hybrid nanocomposite on COD removal of different pharmaceuticals.

The ciprofloxacin antibiotic and MB dye solutions are used as an organic pollutant models to estimate the photocatalytic activity of the prepared nanocomposite under solar simulator irradiation, and the results are shown in Fig. 8a,b. In the absence of simulating sunlight, the MB dye adsorption percent by ZnO and CuS QDs @ ZnO nanocomposite is 43% and 54%, respectively, while the ciprofloxacin antibiotic adsorption percent is 41% and 45%. CuS @ ZnO nanocomposite showed the higher adsorption percent than bare ZnO due to its larger surface area. The greater the specific surface area of CuS QDs @ ZnO, increase the electrostatic attraction between waste and PC, and thus the increased the adsorption efficiency^74,75^.

In the presence of sunlight, the total dye removal after 45 min is found to be 82% and 100% for pure ZnO and nanocomposite respectively. In addition, the ciprofloxacin removal is 60% and 100% after 90 min. It can be concluded that the junction formed by adding the CuS QDs to the ZnO NRs improves its photocatalytic efficiency where CuS QDs @ ZnO shows a higher degradation percent than bare ZnO NRs (for MB dye and ciprofloxacin antibiotic). Improving the photocatalytic activity for nanocomposite can be due to the improvement in the crystallite size, the decrease in the bandgap, as well as the reduction of the recombination rate of electrons and holes. As a result, CuS QDs can act as co-catalysts to accept photogenerated electrons from ZnO, significantly improving photocatalytic activity in agreement with^57^.

#### Effect of CuS QDs ratios on the CuS QDs @ ZnO hybrid nanocomposite degradation efficiency

In addition, the effect of the CuS QDs ratios (from 0.5 to 9 wt%) on the degradation efficiency of ciprofloxacin antibiotic has been studied and illustrated in Fig. 8c. It is found that, the bare ZnO degradation efficiency against the ciprofloxacin is 60%. Whereas, it increased to 78%, 87%, and 90% with adding CuS QDs by the ratios 0.5 wt%, 1 wt% and 3 wt%, respectively. Nevertheless, as the CuS QDs content increased to 5 wt%, 7 wt%, and 9 wt%, the degradation efficiency has been reduced to 88.8%, 85%, and 80.0%, respectively. The improvement in the photocatalytic degradation efficiency with adding CuS QDs to 3% is mainly due to increase the adsorptoion efficiency. It is worth mentioning that efficient polluants adsorption and transfer to active sites can contribute to photocatalytic efficiency and is a critical step in the catalytic process because the catalytic process begins at the surface.

In addition to the decrease in the bandgap values, which improved the absorption of light and the production of more e–h pairs. This result is consistent with the UV–Vis spectra shown in Fig. 7a. 3% CuS QDs content showed the highest photocataytic degradation efficiency as it showed highest dispersibility, absorption and lowest band gap. At a higher percentage of CuS QDs, the electrostatic attraction of negatively charged CuS QDs and positively charged holes increases the recombination, thus decreasing the PC reaction efficiency in accordance with^52^. Accordingly, the next discussion will focus on the 3% CuSQDs@ ZnO photocatalyst^73^.

#### Effect of 3% CuS QDs @ ZnO on degradation of different pharmaceuticals and pesticides pollutants

The degradation efficiency of the 3% CuS QDs @ ZnO hybrid nanocomposite photocatalyst against ibuprofen, and ceftriaxone pharmaceuticals, as well as pesticides pollutants namely 2, 4, 5 T, is estimated under the solar simulator irradiation Fig. 8d. The results demonstrate that the PC tends to achieve 100%, 100%, and 30% degradation for ibuprofen, 2,4,5 T, and ceftriaxone after 60 min respectively.

Moreover, the complete degradation of ceftriaxone is achieved after 90 min. The higher degradation efficiency of ibuprofen, 2,4,5 T, and ceftriaxone are attributed to the higher polar surface of these molecules in aqueous. The great polar surface area has increased the capability of adsorption for these wastes over the nanocomposite which, leads to enhancing photocatalytic degradation. Furthermore, the COD of the degradation process has been measured in Fig. 8e, and it is illustrated that there is a 100% removal of COD for ibuprofen, ceftriaxone pharmaceuticals, and 2.4.5 T pesticide after 2 h.

These results confirm the higher capability of CuS QDs @ ZnO for photocatalytic mineralization of pharmaceuticals and pesticide from wastewater.

#### The mechanism of the photocatalytic process over CuS @ ZnO nanocomposites

To get a deeper insight into the origin of the synergy between CuS and ZnO in organic waste degradation and to understand the improved activity of the nanocomposite we have analyzed the mechanism of the photocatalytic process. For this purpose, Herein, for explanation the band alignment, theoretically the valence band edge potential (E_vb_) and the conduction band edge potential (E_cb_) of semiconductor material can be calculated by using the following equation: of CB and VB band edge position for CuS @ ZnO hybrid nanocomposites^76,77^:

4 $${E}_{CB}=X-{E}_{e}-0.5{E}_{g}$$

5 $${E}_{VB}=X-{E}_{e}+0.5{E}_{g}$$

**Figure 9:**
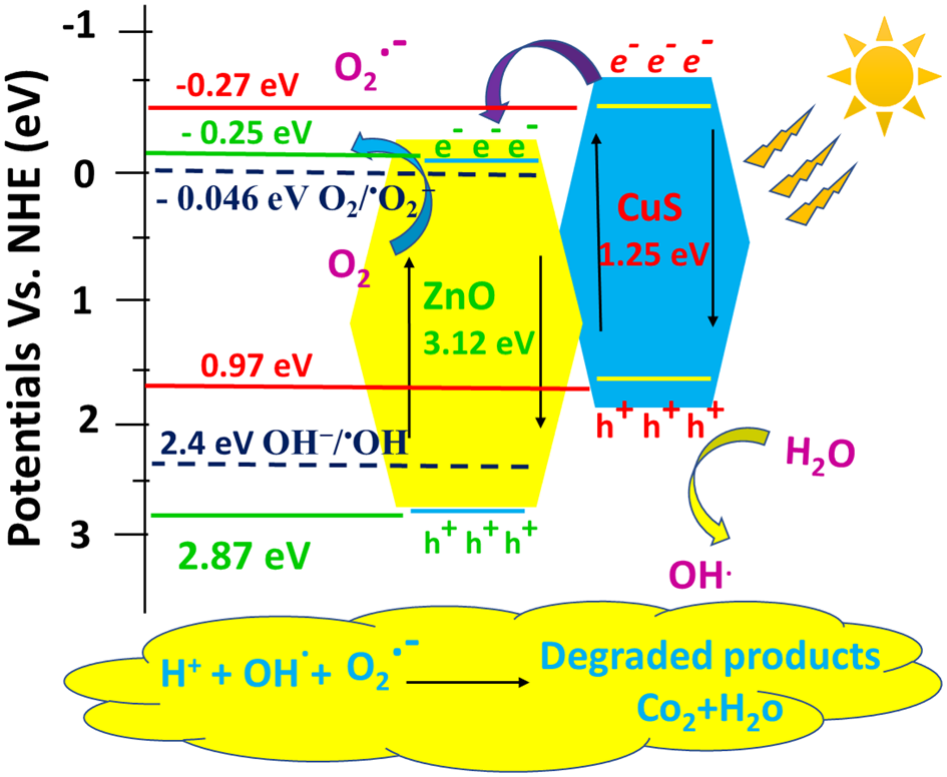
Proposed diagram for the mechanism of photocatalytic degradation of organic wastewater above CuS QDs @ ZnO hybrid nanocomposite under simulated solar light irradiation.

E _g_, E _CB_, E _VB_, refers to the band gap energy The E _g_ of ZnO nanorods and CuS QDs are measured from UV–Vis DRS spectrum with Tauc plot as 3.12 eV and 1.25 eV, respectively, conduction band potential and valence band potential, respectively and E e is the energy of free electrons on the hydrogen scale (4.5 eV) and χ is the absolute electronegativity of the semiconductor, which is determined as the geometric mean of the electronegativity of the constituent atoms (*X* values for ZnO and CuS are 5.81 eV and 4.85 eV^78,79^, respectively). Based on the above formula, the calculated conduction band potential of ZnO is found to be of − 0.25 eV so less negative than that of CuS (− 0.27 eV). The corresponding valence band edge potential estimated for ZnO is of 2.87 eV (vs. NHE) so more positive than that of CuS 0.97 eV (vs. NHE) as illustrated from Fig. 9^79^. The above findings revealed the formation of a heterojunction photocatalyst with a geometric band structure that is favourable to charge carrier separation and transfer.

When nanocomposite is exposed to the simulating solar irradiation, the electrons can be created from CuS QDs when the photon energies of the illuminations exceed their band gap energies. Based on the estimated band edges positions, one can expect that the photo-excited electrons formed in the conduction band of CuS should be transferred to the conduction band of ZnO. Simultaneously, the photogenerated holes also must transfer in the reverse direction, from the ZnO valence band to the CuS valence band. Such a transfer of charge carriers would lead to accumulation of photogenerated holes in CuS and photo-excited electrons in ZnO. In view of this observation, one can conclude that, the ZnO NRs and CuS QDs exhibit suitable band potentials, consequently, the two semiconductors can form heterojunction to enhance the electron transfer and reduce the photoinduced electron–hole recombination as confirmed from PL results^35,62^.

As mentioned above the photogenerated electrons in the CB of CuS QDs can easily transfer to the CB of ZnO NRs. These electrons can react with dissolved O_2_ to produce ^**·**^O_2_^−^ radicals as the CB edge potential of ZnO NRs (− 0.25 eV) is more negative than the potential of O_2_/^**·**^O_2_^−^ couple (− 0.046 eV). The holes retained in the V_B_ of ZnO NRs have a more positive potential than that of OH^−^/^**·**^OH (+ 2.4 eV), therefore, can react with OH^−^ to yield ^**·**^OH^53^. Moreover, the holes in the V_B_ of CuS cannot oxidize OH^−^ to generate ^**·**^OH radicals because of its more negative V_B_ potential (+ 0.97 eV), however, can directly participate in the oxidation reaction of organic pollutants molecules. By this means, the reactive active species ^**·**^O_2_^−^, ^**·**^OH, and h^+^ can be produced, and take part in the oxidative degradation of organic pollutants. The possible photocatalytic reactions are listed here^52^.

6 $$\mathrm{CuS}+\mathrm{hv}\to \mathrm{CuS}\left({\mathrm{h}}^{+}+{\mathrm{e}}^{-}\right)$$

7 $$\mathrm{ZnO}+\mathrm{hv}\to \mathrm{ZnO }\left({\mathrm{h}}^{+}+{\mathrm{e}}^{-}\right)$$

8 $${\mathrm{e}}^{-}\left(CuS\right)\to {\mathrm{e}}^{-}\left(ZnO\right)$$

9 $$\mathrm{ZnO NRs}\left({\mathrm{e}}^{-}\right)+{\mathrm{O}}_{2} \to {\mathrm{O}}_{2}^{.-}$$

10 $${\mathrm{O}}_{2}^{\cdot-}+{\mathrm{H}}_{2}\mathrm{O}\to {\mathrm{HO}}_{{2}^{+}}^{\cdot}{\mathrm{OH}}^{-}$$

11 $${\mathrm{HO}}_{2}^{\cdot}+{\mathrm{H}}_{2}\mathrm{O}\to {\mathrm{H}}_{2}{\mathrm{O}}_{2}+{\mathrm{OH}}^{.}$$

12 $$\mathrm{ZnO}\left({\mathrm{h}}^{+}\right)+\mathrm{O}{\mathrm{H }}^{-}\to {\mathrm{OH}}^{.}$$

13 $$\mathrm{ZnO}\left({\mathrm{h}}^{+}\right)+2{\mathrm{H}}_{2}\mathrm{O}\to {\mathrm{H}}_{2}{\mathrm{O}}_{2}+2{\mathrm{H}}^{+}$$

14 $${\mathrm{H}}_{2}{\mathrm{O}}_{2}\to 2{\mathrm{OH}}^{.}$$

These photogenerated radicals are very active species in the degradation process which would degrade the dye molecules^80–82^, forming intermediate products that completely break into CO_2_, H_2_O (Eq. 15)

15 $${\mathrm{OH}}^{.}, {\mathrm{O}}_{2}^{.-}+{\mathrm{h}}^{+}+\mathrm{pollutant\, molecules }\to {\mathrm{CO}}_{2}+{\mathrm{H}}_{2}\mathrm{O}$$

## Conclusion

In the present work, CuS QD @ ZnO hybrid nanocomposites have been synthesised using a microwave-assisted hydrothermal method as a green preparation process. The aim is to improve the performance of the ZnO NRs photocatalyst for the degradation of dyes, pharmaceuticals, and pesticide degradation of emerging pollutants in water under simulated sunlight (SL). XRD data of CuS QDs shows pure hexagonal phase covellite with high crystallinity and a smaller sphere size of 2 nm, which is confirmed by SEM and TEM results. It is found that the crystallinity and light absorption as well as degradation activity increase with increasing the ratio of CuS to 3% and then decrease with further increase. The 3% CuS QDs @ ZnO hybrid nanocomposite has capability for reduction the electron–hole recombination rate, which enhances degradationm rate of organic pollutants. Its band gap decreases from 3.1 for ZnO NRs to 2.90, which improves light absorption and the generation of electron–hole pairs. 3% CuS QDs @ ZnO hybrid nanocomposite showed efficient photocatalytic activity for degradation of ibuprofen and ciprofloxacin as pharmaceutical and 2.4, 5 T pesticide as emerging pollutants models. The results showed improvement in the activity of the 3% CuS QDs @ ZnO hybrid nanocomposite comparing to bare ZnO nanorods for degrading pharmaceuticals in wastewater. Briefly, CuS QDs@ZnO hybrid nanocomposites are promising and cost-effective photocatalytic materials under simulated sunlight (SL) for wastewater decontamination from pharmaceuticals and pesticides.

## Data Availability

All the data and materials applied in the study could be available from the corresponding author only on academic or other non-business requests.
